# Effect of Graphene Oxide Synthesis Method on Properties and Performance of Polysulfone-Graphene Oxide Mixed Matrix Membranes

**DOI:** 10.3390/nano9050769

**Published:** 2019-05-19

**Authors:** Safae Sali, Hamish R. Mackey, Ahmed A. Abdala

**Affiliations:** 1 Division of Sustainable Development, College of Science and Engineering, Hamad Bin Khalifa University, Qatar Foundation, PO Box 34110, Doha, Qatar; ssali@mail.hbku.edu.qa; 2 Chemical Engineering, Texas A&M University at Qatar, Education City, PO Box 23874, Doha, Qatar

**Keywords:** microfiltration, oil separation, polymeric membrane, nanofiller, polymer composite, flux, hydrophilicity, functional group, carbon to oxygen ratio

## Abstract

Graphene oxide (GO) has shown great promise as a nanofiller to enhance the performance of mixed matrix composite membranes (MMMs) for water treatment applications. However, GO can be prepared by various synthesis routes, leading to different concentrations of the attached oxygen functional groups. In this research, GO produced by the Hummers’, Tour, and Staudenmaier methods were characterized and embedded at various fractions into the matrix of polysulfone (PSf) and used to prepare microfiltration membranes via the phase inversion process. The effects of the GO preparation method and loading on the membrane characteristics, as well as performance for oil removal from an oil-water emulsion, are analyzed. Our results reveal that GO prepared by the Staudenmaier method has a higher concentration of the more polar carbonyl group, increasing the membrane hydrophilicity and porosity compared to GO prepared by the Hummers’ and Tour methods. On the other hand, the Hummers’ and Tour methods produce GO with larger sheet size, and are more effective in enhancing the mechanical properties of the PSf membrane. Finally, all MMMs exhibited improved water flux (up to 2.7 times) and oil rejection, than those for the control PSf sample, with the optimum GO loading ranged between 0.1–0.2 wt%.

## 1. Introduction

Increased water scarcity is becoming more imminent, due to factors such as growing population, urbanization, industrialization and agriculture, as well as impacts of global climate change. Recent surveys conducted by the United Nations Educational, Scientific and Cultural Organization (UNESCO) estimate that 1.8 billion of the population will lack access to fresh water by 2025 [1]. Other studies conducted by the United Nations (UN) estimate that the yearly global water demand will increase by 20–30% by 2050 [2]. To manage these challenges, membrane-based water treatment solutions have been promoted as an environmentally-friendly, low-cost technology for the filtration and purification of non-potable water resources.

Polymeric membranes are the most prevalent membranes in the water treatment and filtration industry due to their flexibility, light weight, low energy requirements, high rejection in comparison to inorganic membranes and low cost of production [3,4]. However, polymeric membranes suffer from fouling, leading to a decline in performance over time, and subject to a trade-off between permeability and selectivity [5]. Membrane modification is one solution to overcome the disadvantages of polymeric membranes.

In mixed matrix membranes (MMMs), this can be achieved through the introduction and dispersion of nanoparticles into the membrane matrix to enhance their properties and performance. MMMs are asymmetric composite membranes made of an organic polymer and inorganic filler [6]. MMMs retain the lower cost and ease of fabrication of the polymeric membrane, but offer enhanced performance [7]. The high separation efficiency, low cost and ease of synthesis provided by the polymers are synergistically enhanced mechanically and chemically from properties attributed to the nanofillers [8], leading to an improved membrane hydrophilicity [9], modified surface structure [10], increased mechanical strength and enhanced anti-fouling properties [4]. Due to the benefits of MMMs, they are more prevalent in the market than their thin film composite counterparts, except for niche applications [11].

Several inorganic nanomaterials have been tested as nanofillers for membranes, such as silver [12] and titanium oxide [13], owing to their small nanoparticle size and anti-bacterial properties. The use of carbon nanomaterials such as graphene and carbon nanotubes (CNT) [14] have also been explored. All these experiments have provided promising enhancements to the properties and performance of the membranes. However, the production of metal, pure graphene and CNT doped membranes is costly [15]. A number of lower cost graphene-like nanomaterials exist, such as graphene oxide (GO) and reduced graphene oxide (rGO). These graphene-based materials share some similar and enhanced properties with graphene, and can be used as a substitute.

GO in particular is a more cost-effective option compared to other carbon-based materials, due the simplicity and timesaving attributes of its synthesis methods, especially for large-scale commercial applications [16]. GO has a combination of advantageous properties, such as a high lateral dimension-to-thickness ratio, an amphiphilic nature owing to its numerous surface functional groups, chemical inertness and exceptional mechanical properties [9,10]. This has led to GO being used in membrane applications, including both gas and water separation [5]. When embedded into MMM, GO increases the hydrophilicity of the membranes, as well as the water flux. Moreover, higher rejection of dyes, oil and salt have been achieved using MMMs containing GO, due to the presence of acidic functional groups that induce negative charge on the membrane surface [10]. GO is also known to have an amphiphilic nature, where water molecules are absorbed onto the hydrophilic sites, and then diffuse onto the hydrophobic carbon sites, resulting in water channels [17]. As such, GO has received significant attention as a nanofiller in MMMs, and has demonstrated promising results in a number of studies [9,18,19].

GO is synthesized from graphite in two steps. In the first, graphite flakes are oxidized to graphite oxide, leading to the introduction of oxygen-containing functional groups, such as epoxy (C–O–C), hydroxyl (OH), carbonyl (C=O) and carboxyl (R–COOH) groups into the basal plane and the edges of graphene oxide sheets [11,16]. In the second step, the produced graphite oxide is readily exfoliated in water assisted by sonication or shearing to produce GO suspension [20]. Various methods are used to prepare graphite oxide from graphite by varying the oxidizing agents and intercalants employed, producing GO with varied characteristics and structural properties. The Brodie method, that was developed in 1859, is the first method to produce graphite oxide by adding KClO_3_ to a mixture of graphite and fuming HNO_3_ [21]. Later, the Staudenmaier method was developed based on the Brodie method, where a mixture of H_2_SO_4_ and HNO_3_ is used as the intercalant, producing graphite oxide with a higher C/O ratio. However, these two methods rely on a lengthy oxidation step that takes up to one week. Today, the most widespread GO synthesis method is the Hummers’ method, developed in 1958 [22]. In this method, H_2_SO_4_ is used as the intercalant and NaNO_3_/KMnO_4_ as the oxidizing agents and the oxidation step is completed within 2 h. However, the Hummers’ method has been criticized because of the release of toxic gases such as NO_x_ and ClO_2_ into the environment. More recently, an improved Hummers’ method was developed by Tour by eliminating NaNO_3_, and using a 9/1 mixture of H_3_PO_4_ and H_2_SO_4_. The Tour method does not only eliminate the production of toxic gases, but also produces a more oxidized graphite oxide with a more regular carbon framework and larger sheet size [16].

A number of researchers have evaluated the impacts of GO prepared via Hummers’ or modified-Hummers’ (removing or replacing NaNO_3_) on polymeric membranes, as they are the fastest methods to produce GO [10,18,23]. However, the impact of other GO synthesis methods on the performance and properties of MMM has not been systematically evaluated. Each of the synthesis methods is distinct in the chemicals and conditions used for the oxidation process, resulting in differences in the degree of oxidation and the ratio of the different functional groups present, thereby impacting the GO overall properties. Therefore, this paper investigates the influence of various GO fabrication methods (Staudenmaier, Hummers’ and Tour) on the properties and performance of polysulfone MMMs. In addition, the study investigates the role of GO concentration in the MMM to find whether interactions exist between synthesis method and optimum GO loading.

## 2. Materials and Methods

### 2.1. Materials

Polysulfone (PSf), with an average molecular weight of 35,000 mol, polyvinylpyrrolidone (PVP), with an average molecular weight of 55,000 mol, and N-Dimethylacetamide (DMAc), were all purchased from Sigma Aldrich, St. Louis, MO, USA. PSf was chosen due to its chemical stability and mechanical properties, as well as its ability to withstand higher temperatures compared to other polymers [9]. GO_H_ (a specific form of graphene oxide (GO)) was prepared by the Hummers’ method [22] and was purchased from Sixth Element Materials Technology, Changzhou, China. GO_S_ was prepared following the Staudenmaier method [24], and GO_T_ was prepared by following the Tour method [25] using 45 μm natural graphite flakes kindly supplied by Asbury Carbons, Asbury, NJ, USA. Diesel was obtained from a local petrol station (Woqod) in Doha, Qatar, and was used to prepare the oily-water emulsion of 100 ppm to test the selectivity of membranes.

### 2.2. GO Preparation

GO_S_ was prepared by following the Staudenmaier method [24] where 1 g of 45 µm natural flake graphite (Asbury Graphite) was mixed with 27 mL of an ice-cooled 2:1 H_2_SO_4_:HNO_3_ mixture. The reaction’s temperature was maintained below 35 °C by slowly adding 11 g of KClO_3_. After 96 h, 800 mL of UP water was added to the solution while stirring, and was filtered using 0.45 µm nylon membrane. The precipitate of GO was repeatedly washed with 5% HCl solution, first to remove sulfate ions, which were detected using the BaCl_2_ test, and then washed repeatedly with UP water to remove chloride ions, which were detected used the AgNO_3_ test.

GO_T_ was prepared via the Tour method [25], which consists of adding 1 g of 45 µm natural flake graphite (Asbury Graphite) to a mixture of 9:1 of H_2_SO_4_:H_3_PO_4_ mixture for 3 min while stirring. Then, 5 g of KMnO_4_ was added to the solution. The reaction’s temperature was maintained at 20 °C for three days. The mixture was centrifuged at 5000 rpm for 30 min and then spent sulfuric acid was decanted. The last step consisted of washing the graphite oxide particles with UP water six times, and centrifuging it at 5000 rpm for 60 min.

### 2.3. GO Characterization

The structure and composition of the three GO samples were analyzed by X-ray powder diffraction (XRD) (D8 Advance, Bruker, Billerica, MA, USA) using CuKα radiation. The intensity graphs displayed were used to evaluate the interlayer distance by solving Bragg’s Law [21]. X-ray photoelectron spectroscopy (XPS) (Escalab 250 Xi, Thermo Fisher Scientific, Waltham, MA, USA) was used to analyze functional group composition with a pass energy of 20 eV for narrow scans and 100 eV for surveys.

### 2.4. Membrane Fabrication

The PSf/GO ultrafiltration mixed matrix composite membranes (MMMs) were prepared via the phase inversion method [26]. Required amounts of GO corresponding to loadings of 0, 0.05, 0.1, 0.2, 0.4 and 0.8 wt% relative to PSf were dispersed by sonication in the DMAc for 5 min under 2 s on, 3 s off, pulsing. Then, solutions of 15% PSf, 5% PVP, and 80% N-DMAc/GO were prepared by stirring overnight. The PSf/GO-nanocomposite solution was first degassed for 10 min in a sonication bath (Ultrasonic cleaner, Cole-Parmer, Vernon Hills, IL, USA), and a 200 µm film was cast on a glass plate using a membrane casting machine (Porometer MEMCAST, Convergence, Enschede, The Netherlands). After 30 s, the glass plate was immersed in a room temperature coagulation bath containing ultrapure (UP) water for 5 min. The coagulated membrane film was then stored in UP water with a daily change of the water for at least three days before testing. For membrane testing and characterization, the membrane film was cut into 47 mm disks and 5 mm wide rectangles using die cutters.

### 2.5. Membrane Characterization

The membrane hydrophilicity was analyzed by measuring the contact angle in a drop shape analyzer (DSA25, Kruss, Hamburg, Germany) following the sessile drop method and Young’s Laplace calculations. Each prepared membrane was tested three times, and the first eight values of each experiment were averaged for the contact angle measurement. The membranes’ surface charge was analyzed at pH 5.2 using a zeta potential analyzer (SurPASS™ 3, Anton Paar, Graz, Austria). Atomic force microscopy (AFM) (Dimension Icon, Bruker, Billerica, MA, USA) was used to obtain images of the membrane surface, as well as morphological features at three surface areas with square dimensions of 2, 10 and 25 µm. The membrane surface and cross-sectional morphologies were imaged using scanning electron microscopy (SEM) (FEI Quanta 400 SEM, Thermo Fisher Scientific, Waltham, MA, USA), operated at a pressure of 10 Pa with an acceleration voltage of 5 kV. For imaging of the cross-section, thin strips of the membrane were fractured by wetting in water and immersing in liquid nitrogen prior to air-drying the strips and placing them on an SEM stub for gold sputtering. The mechanical behavior of the membranes was evaluated via uni-direction tensile testing using dynamic mechanical analysis (DMA) (DMA Q800, TA Instruments, New Castle, DE, USA). Specimens with dimensions of 5 × 30 cm (W × L) were tested at 25 °C and constant speed of 500 µm/min until breakage. The membrane porosity was determined using the gravimetric method from the relation:

(1) $$p=\frac{m_{1}-m_{2}}{A\times l\times \rho_{W}}$$

where *p* is porosity, *m*_1_ and *m*_2_ are weight of the membrane in the dry and wet state (kg), respectively, *A* is the membrane area (m^2^), l is the membrane thickness (m) and $\rho_{W}$ is water density (998 kg/m^3^). All membrane characterization experiments were repeated on three different membrane areas with the exception of AFM and SEM, which were conducted on a single section.

### 2.6. Membrane Testing: Flux and Oil Rejection

The membrane performance was evaluated by measuring the flux of UP water and 100 ppm oil-emulsion using a dead-end cell (Sterlitech HP4750, Kent, WA, USA) at a trans-membrane pressure of 2 bar. The water and emulsion fluxes (*J*) in L m^−2^ h^−1^ (LMH) were calculated as follows:

(2) $$J=\frac{V\times 60}{t\times A\times 1000}$$

where *V* is the volume of permeated water (mL), *t* is time (min), and *A* is the active membrane surface area (14.6 cm^2^). The oil concentration of the feed, permeate and reject solution was measured using total organic carbon (TOC) in a TOC analyzer (TOC-L, Shimadzu, Kyoto, Japan) for the oil samples before and after permeation by the following equation:

(3) $$R\left(\%\right)=\frac{\left(C_{0}-C_{P}\right)}{C_{0}}\times 100$$

where *R* is the oil rejection and *C*_0_ and *C*_P_ are the feed and permeate concentrations (ppm).

## 3. Results and Discussion

### 3.1. GO Characterization

To probe the extent of oxidation and changes in GO structure due to the differences in oxidation conditions, XRD was used to evaluate the interlayer distance between GO particles as well as the crystal structure. Based on the results summarized in Table 1, all three samples show a sharp intense peak corresponding to the 002 plane at 2θ between 9.7° and 11.5° and disappearance of the graphite 002 peak (2θ = 26.5°), indicating complete oxidation of all the graphite layers leading to the increase in the d-spacing. The interlayer spacing estimated using Bragg’s law reveals that the d-spacing of the GO_T_, GO_H_ and GO_S_ samples is 9.1, 8.7 and 7.7 nm, respectively. These results are consistent with previously reported values [27]. The d-spacing depends on the amount of strongly adsorbed water in the hydrophilic zones of the GO, and therefore increases with the decrease in C/O ratio.

The XPS survey spectrum of the three GO samples confirms that GO is composed mainly of carbon and oxygen, with negligible concentrations of nitrogen that is introduced during the preparation of the GO particles. The deconvolution of the high resolution C1s indicates the presence of sp^2^ carbon (C–C/C=C, 284.8 eV), epoxy (C–O, 286.7 eV), carbonyl (C=O, 288.3 eV), and carboxyl (O–C=O, 289.6 eV) groups. The deconvolution of the O1s spectrum confirms the presence of the C=O (532.8 eV) and C–O (534.4 eV) groups. The results of the elemental analysis and C/O ratio are provided in Table 1, and the XPS survey and high-resolution scans are available in the Supporting Information Figures S1–S3. Based on XPS analysis, the differences among the three GO samples can be summarized as follows:

- GO_H_ has the highest oxygen content and GO_S_ has the lowest oxygen content.
- The carbonyl (C=O) is the dominant oxygen groups in GO_S_ with a content of 28.1%, which is twice or more the carbonyl content of GO_H_ and GO_T_.
- Epoxy is the dominant oxygen groups in GO_H_ (25%) and GO_T_ (21%) compared to 11.5% for GO_S_.
- The results are in accordance with the literature where GO_H_ has a degree of oxidation between 1.7–2.5, while the lowest oxidation degree was achieved by the Staudenmaier method [25,27,28].

The characterization results obtained through XRD and XPS experiments confirmed that the preparation method of GO influences the composition of functional groups. GO_H_ and GO_T_ are prepared using the same oxidizing agent (KMnO_4_), resulting in similar elemental composition and functional groups in comparison to GO_S_, which was prepared by the addition of KClO_3_, and resulted in a significantly lower C/O ratio, and different fractions of the oxygen functional groups [29]. Therefore, it can be concluded that the Hummers’ and Tour preparation methods are more efficient at introducing oxygen groups into the graphite structure due to the use of KMnO_4_ as an oxidant.

Studies have shown that the different synthesis methods result in varying GO sheet sizes, with GO_S_ typically having the smallest sheet size, and GO_T_ the largest. The smaller size of the GO_S_ was confirmed by McAllister et al., who used identical synthesis conditions and graphite flakes to this study. They found GO_S_ particles had dimensions of less than 10 µm, independent of the size of the starting graphite flakes [30]. The average particle size for the GO_H_ was reported to be ranging between 13–14 µm, irrespective of oxidation length under typical preparation conditions [31]. Pan and Moghaddam similarly achieved a particle size of 15.8 μm, which was reduced to 10.4 μm with 10 min of sonication [32]. The same authors showed the Tour method using graphite to KMnO_4_ ratios of 1:6 and 1:4 (comparable to the 1:5 used in this study) produced GO_T_ with particles of 33.8 and 55.7 μm, respectively.

### 3.2. Membrane Characterization

#### 3.2.1. Membrane Hydrophilicity and Surface Charges

**Figure 1:**
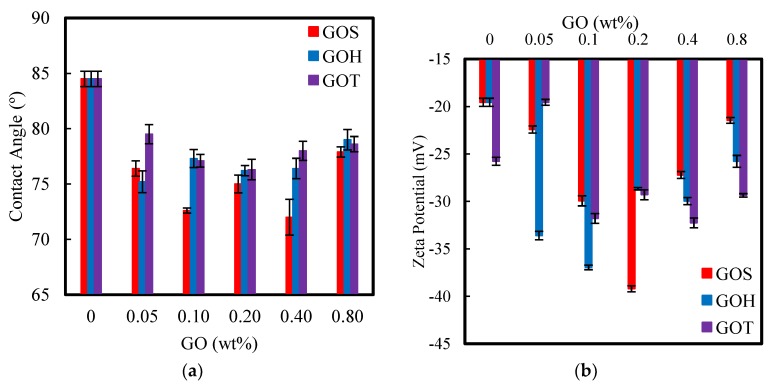
Contact angle (**a**) and zeta potential (**b**) of GO_S_, GO_H_ and GO_T_ membranes. Error bars indicate standard deviation.

Membrane hydrophilicity is a key parameter, that impacts not only the membrane performance in terms of flux and rejection, but also the membrane antifouling characteristics. Hydrophilicity of the membranes was evaluated by water contact angle measurements. Regardless of the synthesis method, GO increased the hydrophilicity (lower contact angle) of the PSf membrane, as shown in Figure 1. Moreover, the three membrane sets show similar patterns where the contact angle decreases as the GO loading increases until reaching a minimum, and then increases as the loading increases further.

The membrane modified with GO_S_ shows the highest hydrophilicity (lowest contact angle of 72°) at 0.4 wt% GO. In all cases, the hydrophilicity showed only minor changes under different GO loading, but all samples yielded significantly higher hydrophilicity than the control membrane. The affinity of the GO nanoparticles and their functional groups with water molecules contributes significantly to the enhanced hydrophilicity. Moreover, these results are further explained by the possible diffusion of GO sheets to the membrane surface during the casting process [23]. The proximity of hydrophilic GO sheets to the surface enhances the ability of the membranes to interact with water molecules making them more hydrophilic.

The small difference in contact angle among the different membrane sets is attributed to the presence of different oxygen functional groups on the surface of GO prepared by different methods. For instance, the carbonyl groups are usually located at the GO edges, and edge-situated functional groups are known to contribute more strongly to hydrophilicity than those situated on the basal plane [33]. Epoxy groups in contrast, are known for their hydrophobicity [34]. Interestingly, GO_S_ modified membranes have the highest hydrophilicity, even though the oxygen content of GO_S_ is the lowest among the GO samples. However, GO_S_ had the highest percentage of carboxyl plus carbonyl groups (42.2%), while the carboxyl and carbonyl content in GO_H_ and GO_T_ were only 29.9% and 32.2%, respectively.

The zeta potential measures the surface charge of membranes, which, in turn, can indicate their rejection performance. A high negative surface charge reveals that the electrostatic repulsion between the membrane surface and the negatively charged ions is high, and therefore, their chance of being removed from water increases. The negative zeta potential value is linked to the presence of oxygenated functional groups (carbonyl, hydroxyl, carboxyl and epoxy groups) on the surface of the membrane [23]. The addition of GO into the membranes’ matrix impacted the surface charge of the membrane significantly. As summarized in Figure 1, the zeta potential values of the modified membranes became more negative compared to the control membrane, implying that they have the ability to reject more ions than the control membrane. In concordance with the contact angle results, the GO_S_ set displayed the most negative surface charge with a decrease up to −39.2 mV for the 0.2 wt% membrane. A similar pattern was followed by the other two GO sets. At higher GO loadings, the zeta potential increases towards zero, possibly due to the agglomeration of GO sheets. The aggregation of the nanoparticles can take place during the casting of the membranes because of the strong van der Waals interactions between particles and the functional group dominance [12].

#### 3.2.2. Membrane Surface Structure

**Figure 2:**
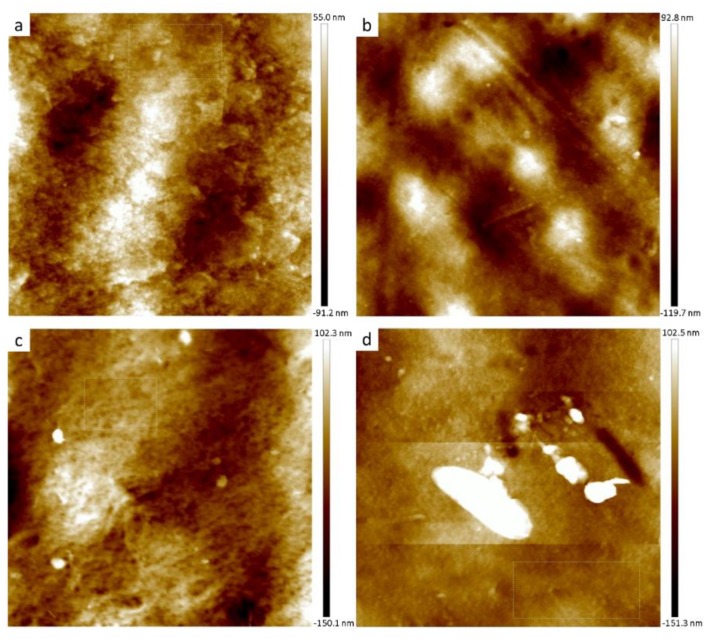
Atomic force microscopy (AFM) surface topography of (**a**) GO-0 wt% (**b**) GO_S_-0.2 wt% (**c**) GO_H_-0.2 wt% and (**d**) GO_T_-0.1 wt% membrane taken at 10 × 10 μm scan area.

**Figure 3:**
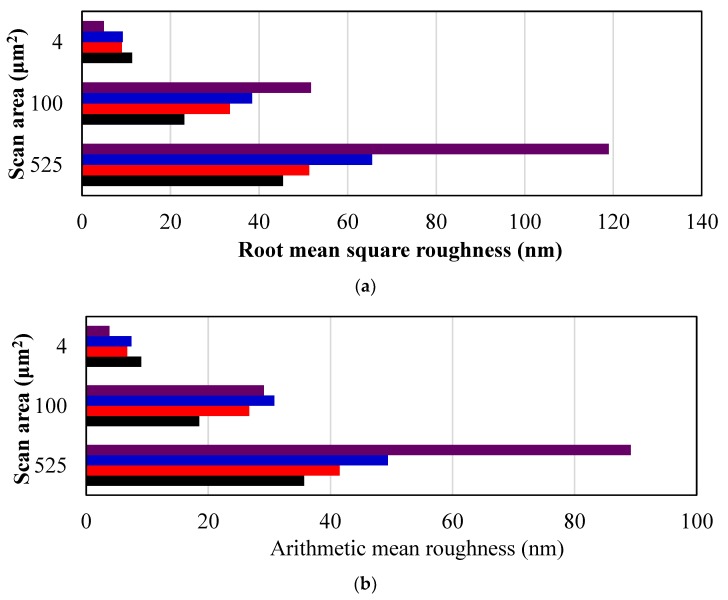

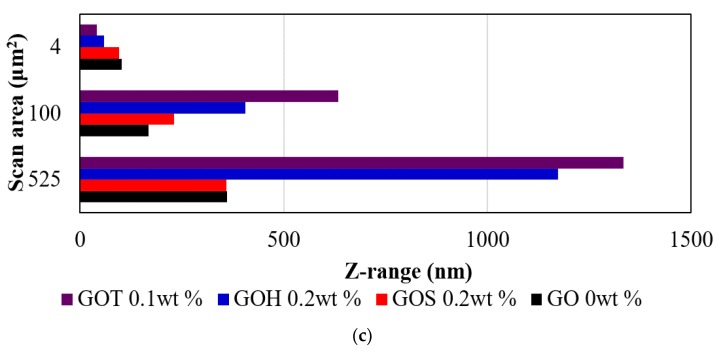
AFM surface roughness analysis of polysulfone-graphene oxide (PSf-GO) membranes with optimum loading of GO_S_, GO_H_, and GO_T_ measured by AFM at various scan scales showing (**a**) root mean square roughness, (**b**) arithmetic mean roughness and (**c**) z-range.

The surface and cross-section morphologies of the membranes influence the permeability and selectivity of the membranes. Therefore, the surface roughness and surface topography were evaluated via AFM under tapping-mode for membranes that had shown optimal flux performance in each set, and are shown in Figure 2. The GO_T_ 0.1 wt% membrane was used as a reference for the GO_T_ set, as it has displayed enhanced performance and properties compared to the other membranes, while the 0.2 wt% membranes were used for GO_S_ and GO_H_ for the same reason. The GO-embedded membranes showed a ridge-and-valley surface, where the lighter regions represent the highest points of the membrane, and the darker areas represent the lowest points and/or pores of the membrane. At larger scan areas (25 × 25 and 10 × 10 μm) the modified membranes showed a rougher surface than the control membrane as displayed in Figure 3, which was consistent with previous reports of MMMs [18,35]. GO_T_ showed the roughest surface, followed by GO_H_ and then GO_S_. A rough surface is desirable, as an increased roughness value has been linked to an increase in flux [13]. However, at the finest scan area (2 × 2 μm) the localized roughness was lower, with GO_T_ having the smoothest membrane surface. The difference in behavior for GO_T_ at the largest and smallest scan areas could possibly be influenced by the larger GO sheet size associated with the GO_T_ synthesis method. At the 10 × 10 μm scan size GO_H_ had a larger arithmetic mean roughness, while GO_T_ had the larger root mean square roughness, indicating that GO_T_ showed more irregularity in its surface structure roughness. Similar observations to roughness were observed for the largest peak-valley range (z-value) obtained from each scan (Figure 3).

**Figure 4:**
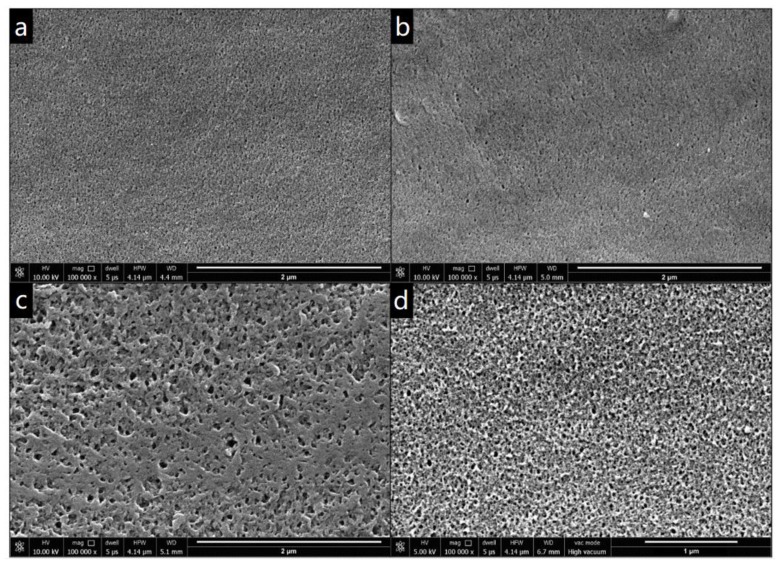
Scanning electron microscopy (SEM) images of membrane surface of (**a**) GO-0 wt%, (**b**) GO_S_-0.2 wt%, (**c**) GO_H_-0.2 wt% and (**d**) GO_T_-0.1 wt% membrane. Scale bars are 2 μm for images a, b and c; and 1 μm for image d.

SEM analysis was conducted on the best performing membranes from each GO synthesis method to understand how differences in synthesis method impact membrane morphology, and therefore performance. The surface images in Figure 4 revealed significant differences in surface structure and porosity between the various samples. The GO-free membrane showed a fine carpet-like surface with fine pores. This same structure was also apparent in GO_S_ doped samples, but with more distinct pore openings. For GO_T_ a more pumice-like surface was present with larger pores, while GO_H_ exhibited the largest pore size with a sponge-like surface.

A lower C/O ratio has been reported to result in an increased stacking of GO sheets, which results in larger channels around the GO stacks [33], consistent with the change in surface roughness and pore size of GO-doped membranes. Nevertheless, the GO nanosheets are not visually apparent on the surface of the membranes, implying that excessive agglomeration did not take place in these particular samples, and the nanoparticles were well dispersed [10]. The surface SEM images are consistent with the AFM surface structures observed at 2 × 2 μm scan area (Supporting Information Figure S4) and AFM measurements at larger scale, corresponding to water drop size, correlated with contact angle measurements, where the roughest surface (GO_T_) had the lowest contact angle. SEM images of the cross-section showed that all the membranes display a typical asymmetric structure, including a thin active layer and large porous support layer, with finger-like structure (Supporting Information Figure S5). It is also observed that more pores are exhibited at the top layer of the cross-section of the GO containing membranes compared to the control membrane.

#### 3.2.3. Mechanical Properties

**Figure 5:**
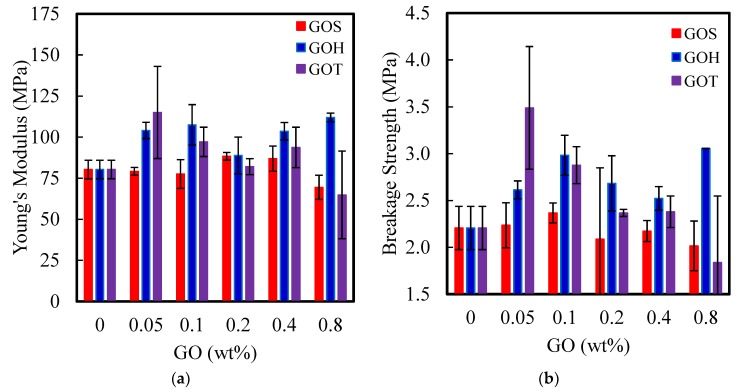
Effect of GO type and loading on (**a**) Young’s modulus and (**b**) breakage strength of GO-PSf membranes. Error bars show sample standard deviation.

The mechanical properties of the pure and modified membranes were examined via tensile testing and the Young’s modulus, and stresses at breakage are summarized in Figure 5. All stress-strain curves exhibited a linear section as an elastic response, followed by a plastic elongation phase before rupturing (Supporting Information Figure S6). The GO_S_ set showed very little variations in mechanical properties with GO loading. The GO_T_ set showed the largest improvement, with an increase in Young’s modulus and rupture stress at low concentrations, but also the largest variation with loading; both properties decreasing notably at the highest GO loadings tested. For the Young’s modulus, two-way independent analysis of variance (ANOVA) showed statistically significant differences between GO_S_ compared to GO_H_ and GO_T_, with Tukey’s post-hoc test giving *p* values of <0.001 and 0.013, respectively. Notable interactions were also observed between synthesis and loading (*p* = 0.026). For stress at breakage, both loading and synthesis methods were significant predictors of behavior, with 2% loading significantly greater than most other loading rates (*p* < 0.001) while for the synthesis method GO_S_ was significantly less than GO_H_ and GO_T_ (*p* < 0.001).

The size of the particles and more specifically the aspect ratio significantly impact the mechanical properties of polymer nanocomposites. Because of that, the small sheet size of GO_S_ does not have any impact on the stiffness of the membranes regardless of the loadings, whereas the large particles of GO_T_ significantly impact the membranes’ stiffness at very low loading, as it is expected to have very low percolation concentration, but stiffness decreases at higher loading due to aggregation of the large GO_T_ sheets. The impact of the surface-to-bulk ratio was previously confirmed by Wu et al. [36]. Moreover, larger particles place larger stress zones in the material, and may be a cause of the increased strength variability observed with GO_T_.

### 3.3. Membrane Performance

#### 3.3.1. Water and Oil Flux

**Figure 6:**
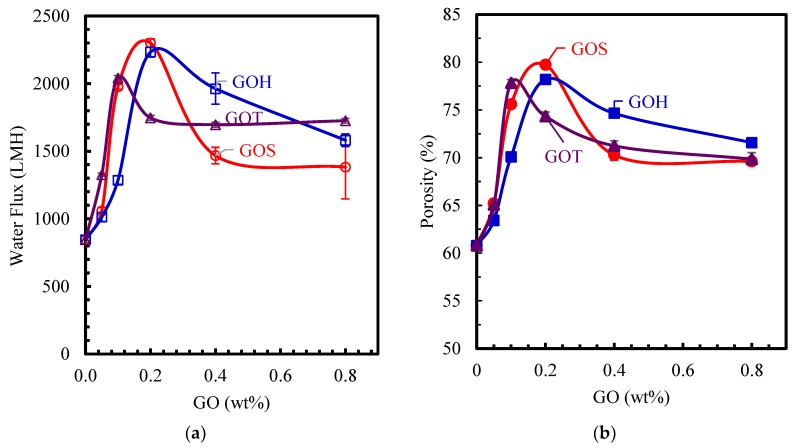
Pure water flux at 2 bar (**a**) and porosity (**b**) of GO modified membranes. Error bars show sample standard deviation.

**Figure 7:**
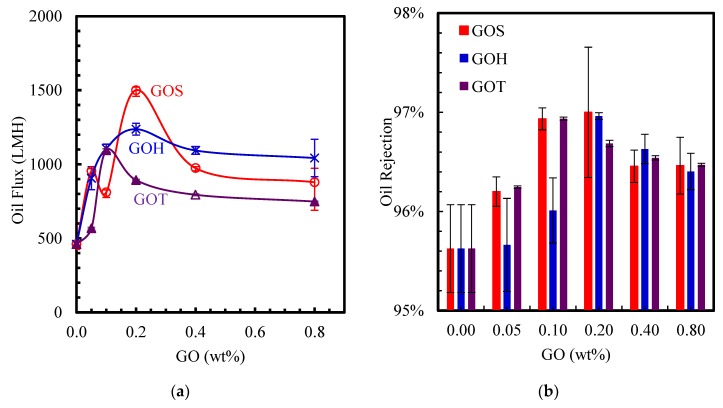
Oil-emulsion flux at 2 bar (**a**) and oil rejection (**b**) of PSf-GO modified membranes.

Permeability testing was conducted by first running UP water in a dead-end cell, followed by an oil-water emulsion at 2 bar pressure. The results of the experiments are summarized in Figure 6 and Figure 7 for water and oil flux, respectively. The control membrane had a stable water flux of 845 LMH and an oily-water flux of 460 LMH. All modified membranes displayed a higher water and oil permeability compared to the control membrane. Generally, all sets of membranes have displayed a trend where the flux increased with increased loading, and then decreased after reaching a peak at the highest loading.

While water and oily-water flux curves showed a consistent and similar trend, the oily-water flux was approximately two-thirds of that achieved with UP water. This decrease of flux is linked to the clogging of the pores by oil particles during the testing. The highest water flux was reached by the GO_S_-0.2 wt% membrane at a value of 2300 LMH. Similarly, the highest oily-water flux was also achieved with GO_S_-0.2 wt% at 1520 LMH. The GO_H_ set followed a similar pattern where the highest flux was achieved by the GO_H_-0.2 wt% membrane for both UP water and oily-water. For the GO_T_ set, the water and oil flux showed a maximum flux at 0.1 wt% loading, which is more than twice the flux of the control sample. At higher loading, flux decreases due to the agglomeration of the GO sheets, thereby reducing the membrane porosity (Figure 6b), and the effectiveness of GO to enhance the hydrophilicity, as confirmed by the contact angle measured and consistent with previous results [37,38]. Moreover, based on the results in Figure 6, it is clear that the membranes doped with GO_H_ and GO_T_ showcase similar trends, while the GO_S_ set is different. This behavior is linked to the type of dominant functional groups in GO and their hydrophilicity. GO_H_ and GO_T_ contain a higher concentration of epoxy groups, while GOs has a large concentration of the more polar carbonyl groups, making GOs more effective at increasing the hydrophilicity [33]. Moreover, Ballinas et al. [39] confirmed that the size of the nanoparticle embedded into the membrane influences the permeation with a smaller nanoparticle size resulting in a higher permeation. Hence, the low particle size of GO_S_, which is linked to the harsh oxidizing, is more effective than GO_H_ and GO_T_ in increasing the porosity and the water flux.

Figure 6b shows the effect of GO loading and synthesis method on membrane porosity measured by the water wicking method. As the loading concentration increases, the porosity of the membranes increase from 60% for the control membrane, to a peak of 77–79% for a GO loading of 0.1–0.2 wt%. Beyond this loading the porosity decreases in agreement with previously reported values for GO-PSf MMMs [37]. These porosity results, in addition to the contact angle and zeta potential results, suggest an increased flux for membranes modified with small GO loading, 0.1 to 0.2 wt%.

The results of the pure water flux experiments are consistent with the impact of the GO type on hydrophilicity and porosity, as explained in the previous section. Permeability is governed by the hydrophilicity of the membranes as well as their porosity. The contact angle results provided by the experiments of this study support an increase in hydrophilicity, which is verified by the flux results. The water flux, porosity, and contact angle have similar correlation with GO type and loading as shown in Figure 1 and Figure 6.

#### 3.3.2. Oil Rejection

The oil separation results are summarized in Figure 7 and they display the increased rejection for all membranes relative to the control membrane sample that exhibited 95.6% oil rejection. Similar to the flux behavior, the rejection initially increases with the GO concentration up to an optimum loading, and decreases at higher loading, regardless of the GO type. Nevertheless, the enhancement of rejection is relatively limited, increasing by roughly 1% only. It is typically expected that flux and rejection are competing objectives, but the use of GO as a nanofiller at appropriate doping concentrations allows improvement in both objectives simultaneously for oil removal, due to the tailoring of surface contact angle and hydrophilicity.

The carbonyl and carboxyl groups are more dominant in the GO_S_ set, which explains the better performance and properties of the previous results in GO_S_. Based on literature that explored the removal capabilities of GO-embedded membranes, the variation in hydrophilicity, zeta potential, as well as the pore size, are considered to be the main factors that influence the selectivity performance of the membrane [23,40,41]. While a membrane with low contact angle and high negative zeta potential displays a high permeability, a membrane with small and tight pores reduces the flux and increases the selectivity [41]. 

The contact angle and zeta potential results provided by the experiments of this study support an increase in hydrophilicity and ion mobility, which is verified by the separation results.

### 3.4. Role of GO Doping Concentration

Many previous experiments were conducted on GO prepared via Hummers’ or modified Hummers’ method, where the nanoparticles were introduced into the matrix of membranes at various concentrations. Experiments conducted on PSf-GO membranes embedded with Hummers’ or modified Hummers’ GO nanoparticles, have all displayed an enhancement of performance and properties compared to the control membranes. Moreover, the permeability of the membranes increases at low loading concentrations and decreases at higher loadings after reaching a peak [18,40,42]. Selectivity experiments conducted by Park et al. on PSf-GO membranes showed that the highest salt rejection was achieved at 0.25% GO loading [40]. Nasseri et al. prepared a similar PSf-GO modified membrane at two concentrations only. An increase in permeability and rejection of bisphenol A was reported at 0.4 wt% loading, but decreased at 1 wt% GO, which was attributed to the lower electrostatic repulsion displayed by the 1 wt% loaded membrane [18]. The discussed experiments found in literature all agree that the GO modified membranes display a peak performance between 0.1 and 0.4 wt% of loading before the permeation performance decreases at higher loadings. The results of this study have shown a water and oil permeation behavior where the highest performance was displayed by 0.2 wt% loading for GO_S_ and GO_H_ membranes and 0.1 wt% loading for GO_T_ membranes, possibly due to its larger size. The GO synthesis method did not influence significantly the GO doping concentration at which optimum conditions were achieved. Moreover, at these low loadings, the doping of GO becomes an economical approach for improved membrane performance, given the tripling in achievable flux with corresponding improved rejection.

### 3.5. Role of GO Synthesis Method

The specific functional groups strongly influence the properties and performance of the membranes. This is important as previous research has concluded that the carboxyl group is the most hydrophilic group, and a dominance of this group would result in higher affinity with water particles [43], although this particular functional group showed relatively limited variance between the synthesis methods in this study (14.1–18.1%).

The different influence of various functional groups can partly be attributed to their location within the GO, in addition to the chemical properties, as epoxy and hydroxyl groups tend to be located on the basal plane, while the carbonyl and carboxyl groups tend to be located on the edge of the GO [44]. GO_S_, which showed the highest flux results, also possess the highest proportion of carbonyl and carboxyl groups combined. In contrast, some studies have demonstrated that a high value of epoxy and hydroxyl groups tends to lower the water permeability, due to the interactions between epoxy and hydroxyl groups and water particles, supporting observations between the differences in fluxes observed in this study, where both GO_H_ and GO_T_ had high epoxy group content. For a similar reason, a low C/O ratio can decrease flux, due to bulk viscous dissipation and spatially extended friction associated with functional groups from the basal plane, and may further explain why the higher C/O ratio GO_S_ resulted in improved flux, despite the lowest carboxyl functional group content [45]. Shi et al. stated that membranes with GO of low oxidation have shown a flux ten times higher than membranes embedded with GO that possess a high oxidation degree [46].

Size of particles has a significant impact upon the performance of the membranes. The smaller the GO nanoparticles are, the better the flux becomes [39]. GO_T_ which had the largest particles also showed the lowest flux, while the GO_S_ set, which had the smallest particle size, displayed the highest flux.

### 3.6. Selection of GO Synthesis Method for Membrane Enhancement

The GO_S_ and GO_H_ sets displayed the highest performance at the loading of 0.2 wt% with relatively similar flux values. The GO_T_, on the other hand, displayed the highest performance at 0.1 wt% with a slightly lower, but comparable flux value and selectivity. In all cases, regardless of the GO synthesis method, flux improvements ranged between 2.4 and 2.7 times the increase in flux for water. Given that the Tour method of GO preparation is a more sustainable and less time consuming method [25], and achieved its highest flux at only half of the amount required by the other evaluated methods, this could be argued to be the most economic and efficient synthesis method for membrane performance enhancement.

## 4. Conclusions

In this study, three sets of GO prepared via the Staudenmaier, Hummers’ and Tour methods have been characterized and introduced into the matrix of a PSf membrane at various concentrations from 0.05 to 0.8 wt%. In general, the addition of GO nanoparticles into the membrane matrix has helped develop membranes with significantly enhanced properties, compared to the control membrane with the optimal GO loading value of the prepared PSf-GO membranes laying between 0.1 and 0.2 wt%. Key properties defining flux and selectivity performance, such as contact angle, zeta potential and porosity, were all strongly dependent on the GO loading and synthesis type. One membrane from each set of GO modified membranes prepared stood out as exhibiting the best permeation and rejection results, which was at a loading of 0.2 wt% for both GO_S_ and GO_H_ sets and 0.1 wt% for the GO_T_ set. The GO_S_ synthesis method provided the membrane with the highest flux, with an increase of 2.7 times that of the non-modified membrane and best rejection of 97.0%, based on the higher C/O ratio and increased carbonyl content of the GO_S_ synthesis method. While the GO_S_-0.2 wt% membrane displayed the highest flux and rejection, the GO_T_-0.1 wt% membrane displayed a comparable permeability and selectivity at half of the GO_S_ loading requirements. This, in combination with its more environmentally friendly synthesis route, indicates this less commercialized GO synthesis method could be a more sustainable approach to membrane modification.

## Figures and Tables

**Table 1:** X-ray photoelectron spectroscopy (XPS) atomic composition and X-ray diffraction (XRD) interlayer spacing for graphene oxide (GO) variations; GO_S_, GO_H_ and GO_T_.

	Atomic % ^1^		
	GO_S_	GO_H_	GO_T_
C1s C–C/C=C	14.8	7.1	10.4
C1s C–O (epoxy)	11.5	**25.3**	**20.5**
C1s C=O (carbonyl)	**28.4**	11.3	14.6
C1s O–C=O (carboxyl)	14.2	17.3	16.9
O1s C=O	22.1	23.0	26.8
O1s C–O	9.0	11.7	6.7
C/O Ratio	2.2	1.8	1.9
GO interlayer spacing (Å)	7.7	8.7	9.1

^1^ The functional group values obtained from XPS were normalized to omit the nitrogen values which did not exceed 1.2% in any sample.

## Supplementary Materials

The following are available online at https://www.mdpi.com/2079-4991/9/5/769/s1, Figure S1: XPS spectra results of GO_S_, Figure S2: XPS spectra results of GO_H_, Figure S3: XPS spectra results of GO_T_, Figure S4: AFM images of membranes at 2 × 2 μm scan area, Figure S5: SEM images of cross-sections, Figure S6: Stress-strain curves of uniaxial tension tests.
