# Supplementary material for: Effect of Graphene Oxide Synthesis Method on Properties and Performance of Polysulfone-Graphene Oxide Mixed Matrix Membranes
Source: Nanomaterials (Basel). 2019 May 19;9(5):769. doi: 10.3390/nano9050769 (PMC6566723; doi:10.3390/nano9050769)
Supplement: Supplementary file 1 [file nanomaterials-09-00769-s001.pdf]

Effect of graphene oxide synthesis method on properties and performance of polysulfone-graphene oxide mixed matrix membranes

Safae Sali [a], Hamish R. Mackey [a,*], Ahmed A. Abdala [b,*]

[a] Division of Sustainable Development, College of Science and Engineering, Hamad Bin Khalifa University, Qatar Foundation, Doha, Qatar.

[b] Chemical Engineering, Texas A&M University at Qatar, Education City, Doha, Qatar.

[*] co-corresponding authors: hmackey@hbku.edu.qa; ahmed.abdala@qatar.tamu.edu

Supporting Information Document

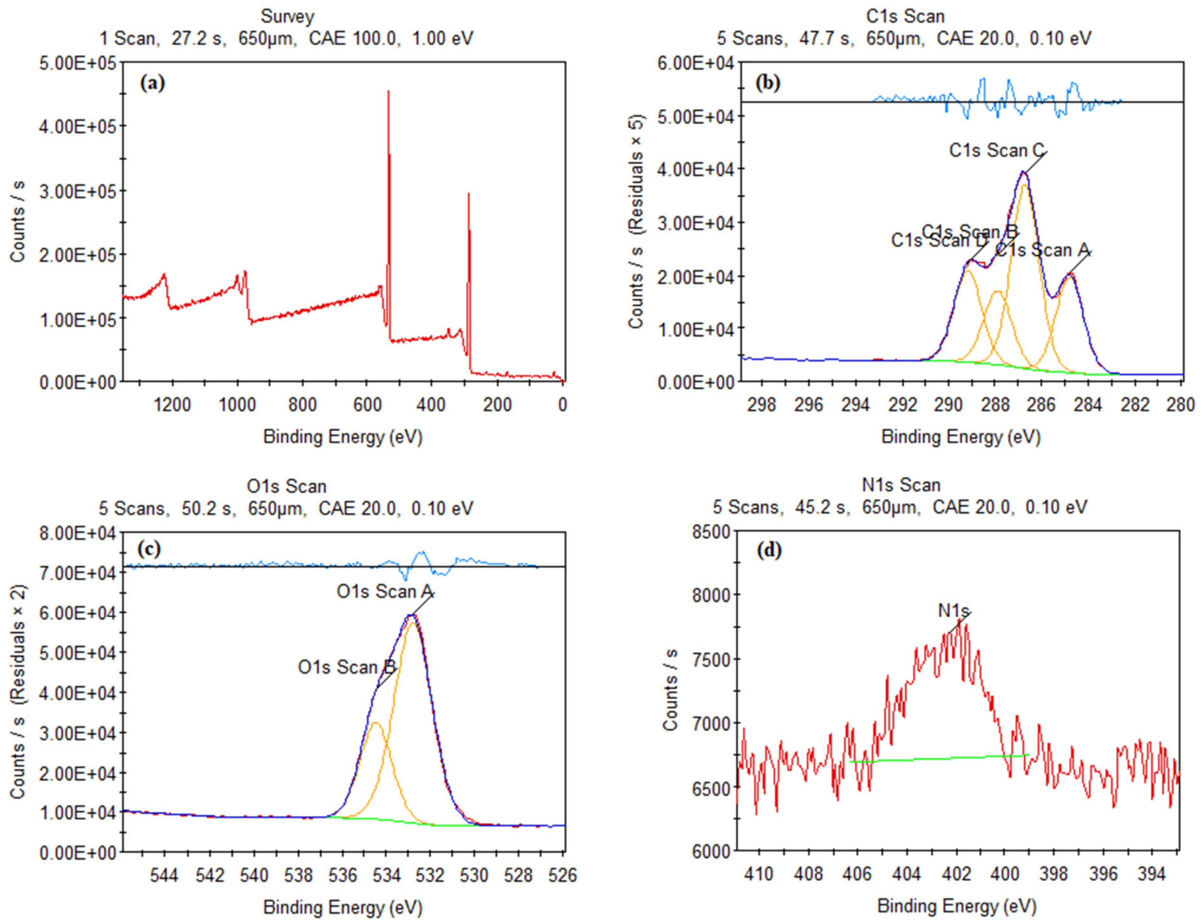

Figure S1 XPS spectra results of GO*S* (a) survey spectrum (b) C1s spectrum (c) O1s spectrum and (d) N1 spectrum

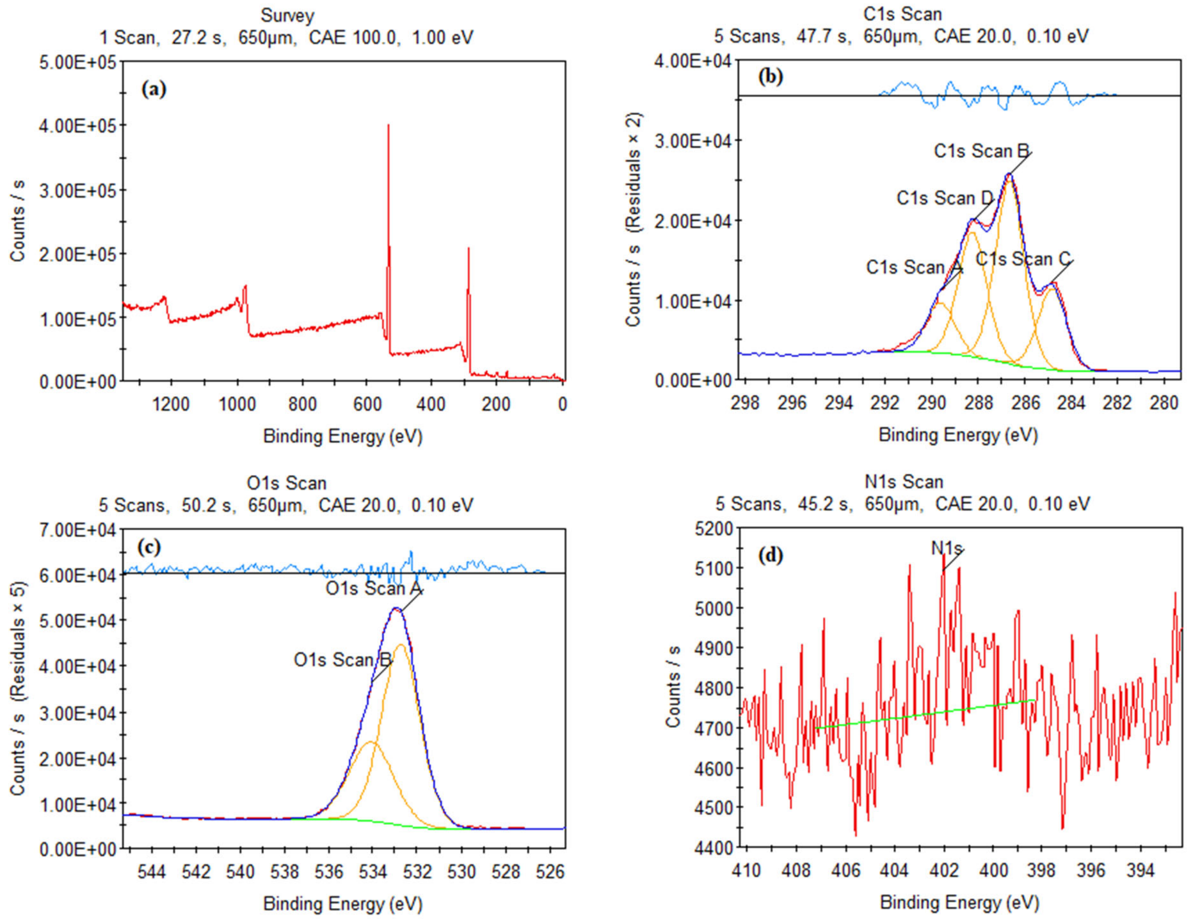

Figure S2 XPS spectra results of GO_H (a) survey spectrum (b) C1s spectrum (c) O1s spectrum and (d) N1 spectrum

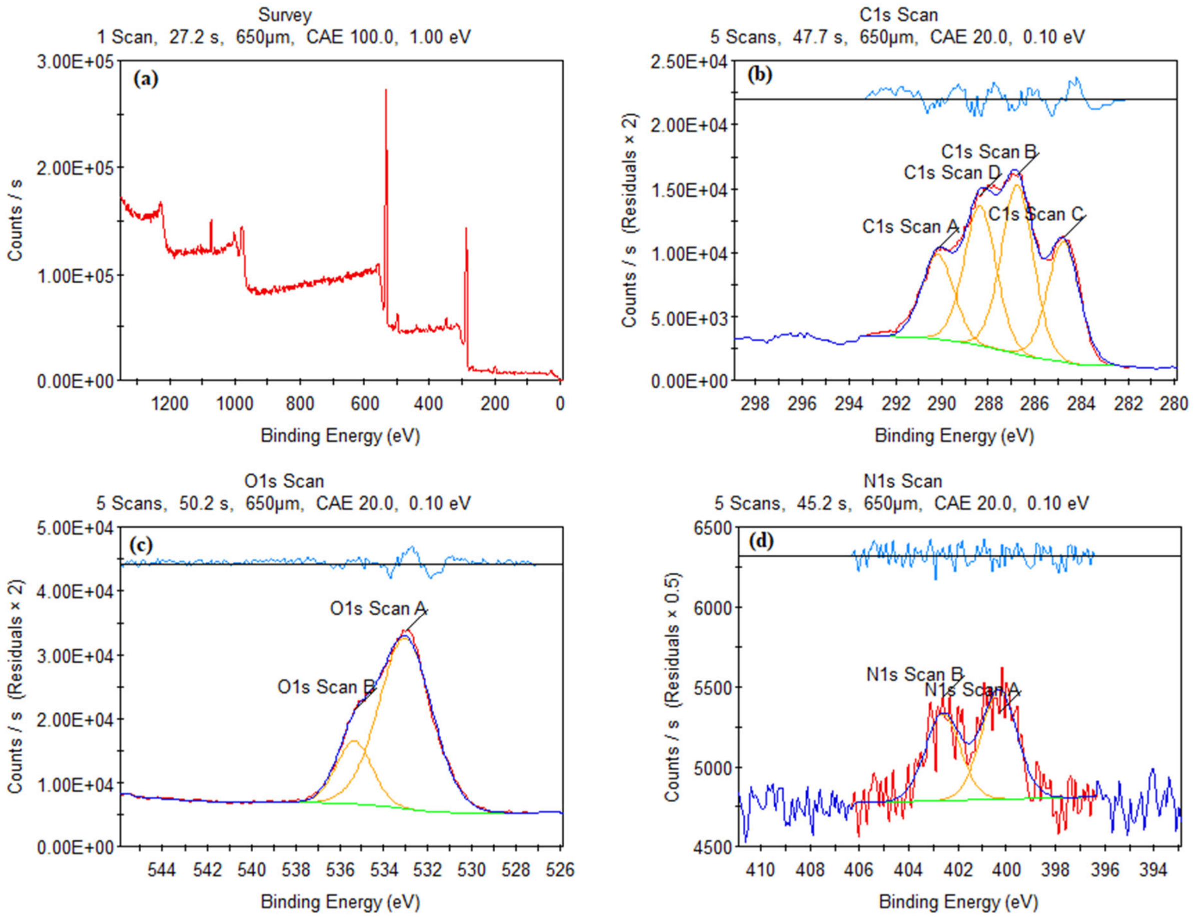

Figure S3 XPS spectra results of GO_T (a) survey spectrum (b) C1s spectrum (c) O1s spectrum and (d) N1 spectrum

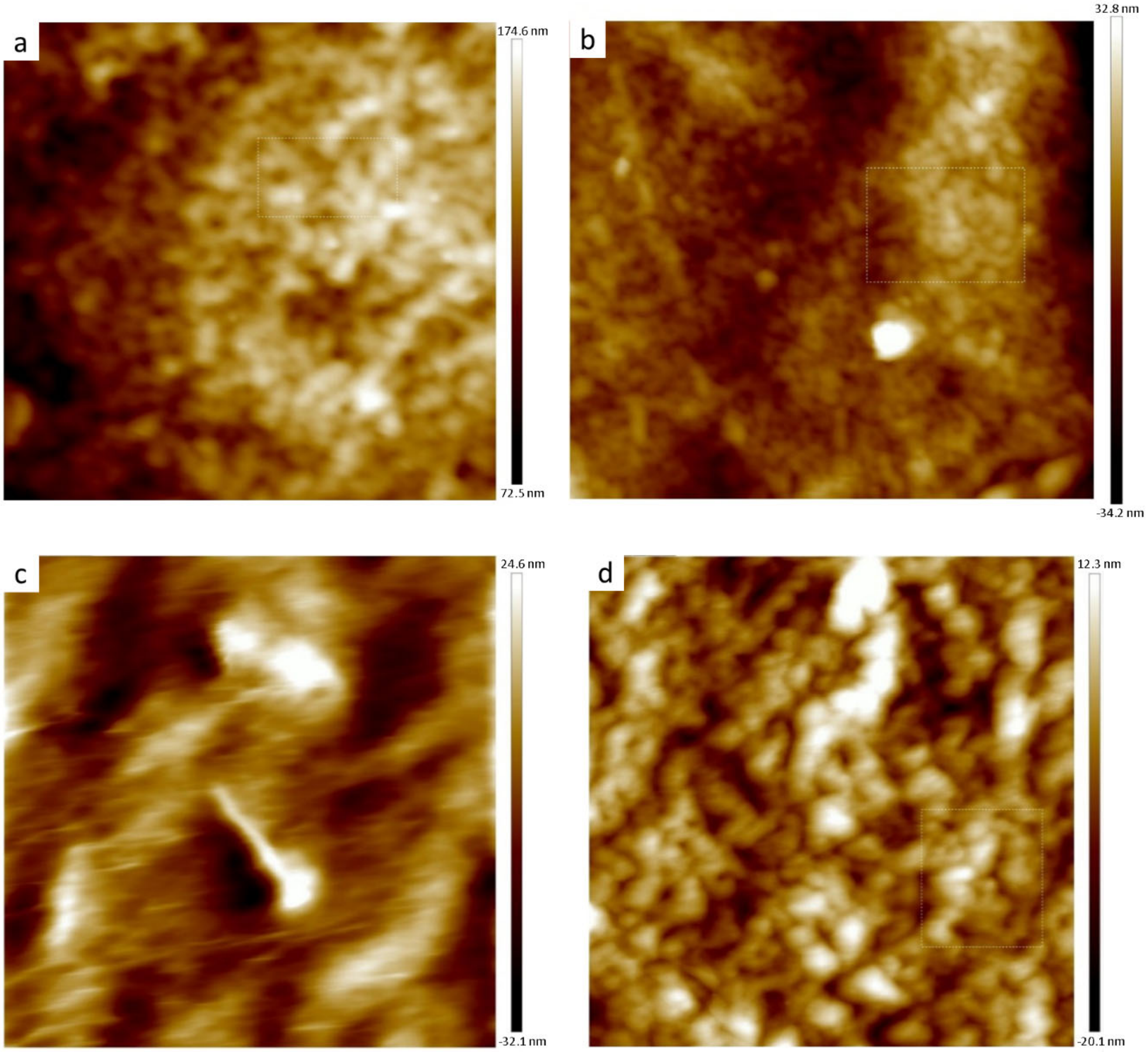

Figure S4 AFM images of membranes at 2×2 μm scan area. a) GO-0wt%; b) GO_S-0.2wt%; c) GO_H-0.2wt%; d) GO_T-0.1wt%.

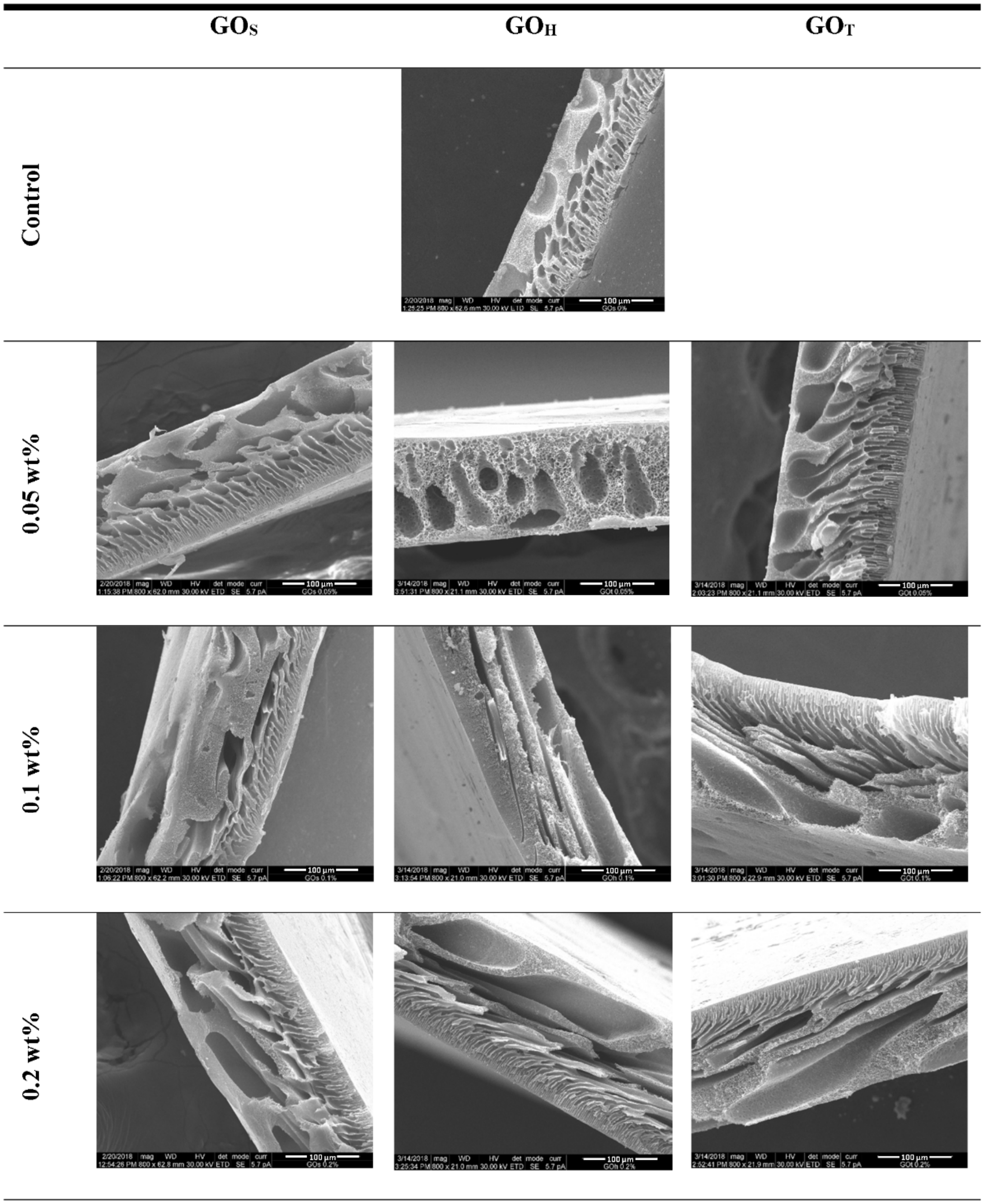

Figure S5 SEM images of cross-section of GO_S, GO_H and GO_T at various concentrations

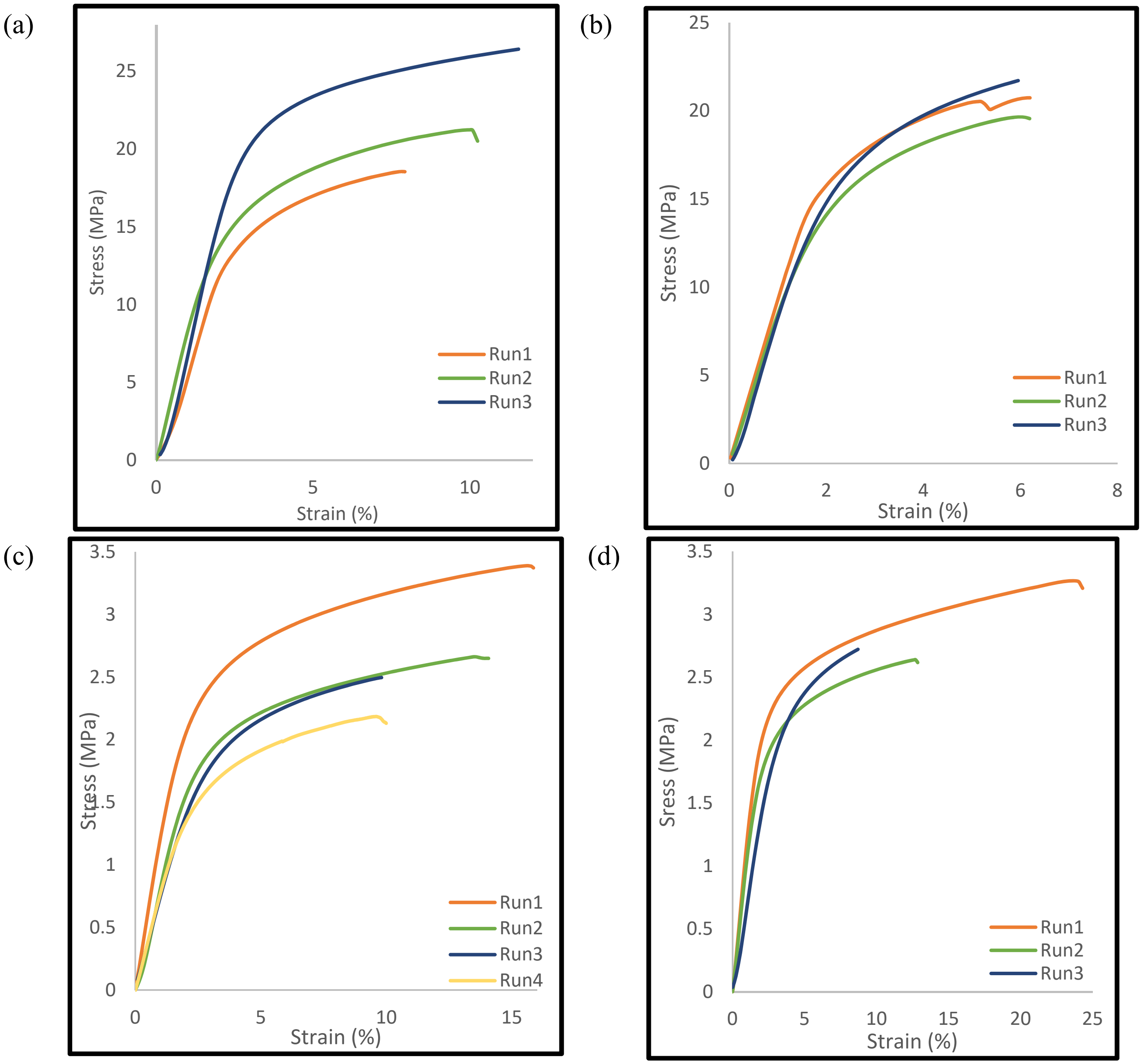

Figure S6 Stress-strain curve of uniaxial tension test for (a) control; (b) 0.2 wt.% GO_S; (c) 0.2 wt.% GO_H; and (d) 0.1 wt.% GO_T
